# Most Antidepressant Use in Primary Care Is Justified; Results of the Netherlands Study of Depression and Anxiety

**DOI:** 10.1371/journal.pone.0014784

**Published:** 2011-03-29

**Authors:** Ellen Piek, Klaas van der Meer, Witte J. G. Hoogendijk, Brenda W. J. H. Penninx, Willem A. Nolen

**Affiliations:** 1 Department of General Practice, University Medical Center Groningen, University of Groningen, Groningen, The Netherlands; 2 Department of Psychiatry, University Medical Center Groningen, University of Groningen, Groningen, The Netherlands; 3 Department of Psychiatry, Neuroscience Campus Amsterdam, VU University Medical Center, Amsterdam, The Netherlands; 4 Department of Psychiatry, Leiden University Medical Center, Leiden, The Netherlands; RAND Corporation, United States of America

## Abstract

**Background:**

Depression is a common illness, often treated in primary care. Many studies have reported undertreatment with antidepressants in primary care. Recently, some studies also reported overtreatment with antidepressants. The present study was designed to assess whether treatment with antidepressants in primary care is in accordance with current guidelines, with a special focus on overtreatment.

**Methodology:**

We used baseline data of primary care respondents from the Netherlands Study of Depression and Anxiety (NESDA) (n = 1610). Seventy-nine patients with treatment in secondary care were excluded. We assessed justification for treatment with antidepressant according to the Dutch primary care guidelines for depression and for anxiety disorders. Use of antidepressants was based on drug-container inspection or, if unavailable, on self-report. Results were recalculated to the original population of primary care patients from which the participants in NESDA were selected (n = 10,677).

**Principal Findings:**

Of 1531 included primary care patients, 199 (13%) used an antidepressant, of whom 188 (94.5%) (possibly) justified. After recalculating these numbers to the original population (n = 10,677), we found 908 (95% CI 823 to 994) antidepressant users. Forty-nine (95% CI 20 to 78) of them (5.4%) had no current justification for an antidepressant, but 27 of them (54.5%) had a justified reason for an antidepressant at some earlier point in their life.

**Conclusions:**

We found that overtreatment with antidepressants in primary care is not a frequent problem. Too long continuation of treatment seems to explain the largest proportion of overtreatment as opposed to inappropriate initiation of treatment.

## Introduction

Depression is a common disorder which is associated with a great amount of morbidity because of its highly recurrent and chronic nature.[1] Most patients with depression are treated in primary care.[2], [3] Guidelines on the treatment of depression in primary and secondary care consider treatment with antidepressants and/or psychotherapy indicated for all patients with major depressive disorder (MDD).[4]–[10] According to these guidelines the treatment should be continued for 6 months after remission (continuation treatment) of a first episode, while it should be continued for one or more years (maintenance treatment) in patients with a recurrent MDD or chronic depression.[4]–[10]

Various studies reported that treatment of depression in primary care is not according to guideline recommendations.[11]–[16] Most studies reported *under*treatment, especially with antidepressants of patients with MDD.[11]–[16] However, in recent years there has also been a lot of attention for *over*treatment with antidepressants.[17]–[19] The fact that in the last 6 months of 2005 760,000 people in the Netherlands (population 16.500.000) were prescribed an antidepressant, according to the Dutch Foundation for Pharmaceutical Statistics (SFK, www.sfk.nl), led to discussions in the Dutch media and among professionals. High numbers were recently also reported in the US and UK, which also led to discussions.[20]–[22] A few studies on *over*treatment with antidepressants suggested that this is mainly due to prescription of these drugs to patients with milder forms of depression, such as dysthymia (Dysth) or minor depression (miD).[17], [18], [23]–[26] Especially the prescription of antidepressants to patients with miD is controversial, as there is no evidence for the efficacy of antidepressants in this condition.[27]–[29] On the other hand, even patients without a depression might receive antidepressants for another justified indication such as anxiety disorders and pain, for which several antidepressants are also registered.

The aim of this study was to evaluate whether the statements reported in the media and a few articles in the literature about widespread overtreatment with antidepressants were true. Therefore, we wanted to assess to what extent the use of antidepressants is in accordance with the Dutch primary care guideline for depression (which is comparable to other international guidelines) as well as for anxiety disorders, with a focus on overtreatment.

## Methods

This study was conducted with baseline-data from the Netherlands Study of Depression and Anxiety (NESDA, www.nesda.nl), a large prospective cohort study on depression and anxiety disorders among respondents aged between 18 and 65 years, recruited from the community, primary care and (secondary) mental health care. Detailed information on the objectives and methods of NESDA were published elsewhere.[30]

### Participants

For this study we selected from the NESDA database only those respondents who were recruited in primary care. In short the recruitment in primary care was as follows. A screening questionnaire was sent to a random sample of 23,750 people from 65 general practitioners (GPs) who had consulted their GP in the past four months, irrespective of reason for consultation. In the Netherlands patients are listed with a single GP or GP practice. The GP is the gatekeeper to secondary care, access to secondary (mental) health care is impossible without a reference from a GP.

The screening questionnaire consisted of the *Kessler-10* (K-10), which has proven screening qualities for affective disorders, and *five additional questions* asking about the presence of specific anxiety disorders. [31], [32] A positive score was defined as a validated K-10 score of ≥20, or a positive score on any of the five anxiety questions.[32] Almost half of the sample (n = 10,706; 45%) returned the screener. Responders to the screener were slightly more often female and older than non-responders.[30], [33] Although having to take small age and sex differences into account, we consider this sample representative of patients consulting their GP in the Netherlands.[33]

Those who screened positive (n = 4592) were approached for a telephone interview with the *short form sections of the CIDI* (CIDI-SF) which has proven screening qualities with a high sensitivity for detecting mental disorders.[34] Specifically trained research staff (mainly psychologists and research nurses) conducted the telephone interview.

All persons who screened positive on the CIDI-SF (n = 898), as well as 196 out of 278 randomly selected persons with a positive K-10 plus, but not fulfilling CIDI-SF criteria and a random selection of 516 screen negatives (healthy controls) participated in the baseline assessment of NESDA (n = 1610), which consisted of a face-to-face interview.

The 79 respondents already receiving treatment for psychiatric conditions in secondary care (defined as more than one contact with either an institute for mental/psychiatric health care or an independent psychiatrist) were excluded from our study sample, yielding a total sample of 1531 respondents for the present analyses.

### Description of Procedures or Investigations undertaken

#### Measures

As part of the screening procedure, all respondents filled out the K-10 plus.

Demographic data (age, gender, ethnicity, education) were assessed during the baseline interview.

Current and lifetime diagnoses of MDD, Dysth, current diagnosis of miD, comorbid anxiety disorders (social phobia, panic disorder, agoraphobia, generalized anxiety disorder) based on DSM-IV were assessed with a structured interview, the *World Health Organization Composite International Diagnostic Interview – lifetime version 2.1 (CIDI)*, which is considered the gold standard for diagnosing depressive and anxiety disorders in large epidemiological studies.[35]–[37] Specifically trained research staff (mainly psychologists and research nurses) conducted the baseline interview including the CIDI.

From the data of the CIDI interview, in which all depressive symptoms were listed separately, we created a variable for *depressive symptoms*, defined as having had one or more DSM-IV symptoms of depression during at least two weeks lifetime with at least either anhedonia or depressed mood, without fulfilling the criteria for diagnosis of MDD or dysthymia. From this data we also created a variable “*chronic MDD*”, defined as having had a lifetime diagnosis of MDD and 24 months of (probably uninterrupted) symptoms of depression in the past five years as recorded with the *life-chart method*. The life chart is a method for recalling depressive symptomatology, the respondent was asked during the interview to mention several important (personal) events from the last several years and was subsequently asked to recall if there was some depressive symptomatology at that point. The life chart has been proven useful to assess the course of illness in patients with mood disorders.[38]–[40]

### Outcome variables

Whether respondents used antidepressants was based on drug container inspection of all drugs used in the past month at baseline and classified according to the World Health Organization Anatomical Therapeutic Chemical (ATC) classification. If respondents had forgotten to take the medication to the interview, their use was based on self-report (done for 35.3% of all subjects). The use of two different methods for assessing antidepressant drugs was not a problem in the current study, as we were not interested in patient compliance, for which self report and drug container inspection can give very different results, but only in physician prescription behaviour. We therefore used the drug container inspection only to assess which medications were used and not for pill counts. Use of antidepressants included selective serotonin reuptake inhibitors (ATC-code N06AB), tricyclic antidepressants (N06AA) and other antidepressants (N06AF/N06AX). St John's wort was not considered an antidepressant.

### Justification for treatment with an antidepressant

To determine the justification for treatment with an antidepressant, we followed the recommendations from the guidelines for depression and for anxiety disorders of the Dutch General Practitioner's Association (NHG). [7], [41] Treatment was considered justified when it was mentioned in the guideline as (one of the) first step option(s) and considered possibly justified when it was mentioned as (one of the) second step option(s). For depressive disorders the depression guideline recommends the use of an antidepressant during six months after response for a first episode of MDD as one of the first step treatment options, although dependent on the degree of suffering or dysfunction. As dysfunction is a criterion for the diagnosis of MDD and patients consulted their physician, we assumed that probably most had at least some degree of suffering or dysfunction. Therefore, we considered treatment with antidepressants justified when a respondent had suffered an episode of MDD in the past year. In case of recurrent or chronic MDD the guideline recommends one to five years of maintenance treatment, with the option for longer in patients with previous recurrences after withdrawal of antidepressants. Therefore, treatment of chronic or recurrent MDD for up to two years was considered justified, all treatment longer than two years was considered possibly justified. In case of dysthymia an antidepressant is mentioned in the depression guideline as (one of the) second step option(s) and therefore considered possibly justified. Antidepressants were not considered justified for depressive states not fulfilling criteria for MDD or dysthymia.

As antidepressants are also registered for the treatment of anxiety disorders, we also considered treatment with antidepressants justified in case the guideline recommendations from the anxiety disorder guideline were followed. This guideline recommends treatment with an antidepressant in case of the presence of an anxiety disorder in the last year, with the option (i.e. possibly justified) to continue the treatment for a longer period.

Overtreatment was considered present when a respondent received an antidepressant without justification or possible justification, i.e. without a non-recurrent (i.e. single) episode of MDD or dysthymia in the past year, or an anxiety disorder or recurrent MDD or chronic depression in lifetime.

### Ethics

The study protocol of NESDA was approved centrally by the Ethical Review Board of the VU University Medical Center and subsequently by local review boards of each participating center. After full verbal and written information about the study, written informed consent was obtained from all participants at the start of baseline assessment. A full ethics statement of NESDA is found elsewhere. [30]

#### Statistical methods

**Figure 1:**
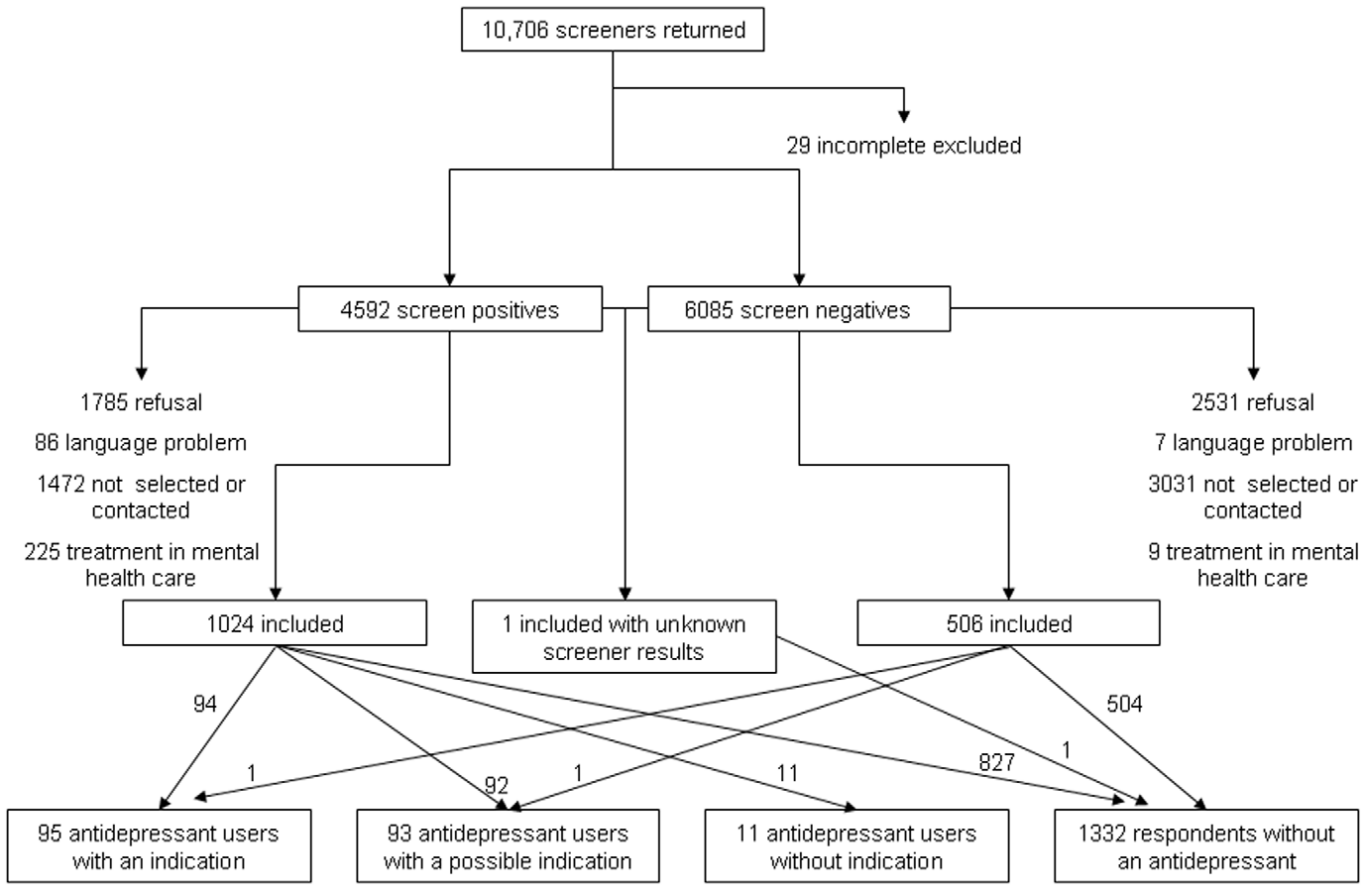
Recruitment flow respondents.

Descriptive statistics and frequencies were used to describe the use of AD and psychological treatment. We recalculated the found numbers and percentages of justified and unjustified treatment with antidepressants in our sample to the original population of 10,677 persons who returned a completed K-10 plus screener questionnaire. This backward projection was done in several steps, which can be derived by reading Figure 1 from the bottom up, or from table 1.

**Table 1:** Results of recalculation of justified and unjustified antidepressant use to screener population.

	Study sample (n = 1531)	Screen positive (n = 4592)	Screen negative (n = 6085)	Screener population (n = 10,677)
Definitely justified AD use	95 (47.7%)	94 * 4.5(1) = 422	1* 12.0(2) = 12	434 (394–474)(3)
Possibly justified AD use	93 (46.7%)	92 * 4.5(1) = 413	1* 12.0(2) = 12	425 (385–465)(3)
Unjustified AD use	11 (5.5%)	11 * 4.5(1) = 49	0 * 12.0(2) = 0	49 (20–78)(3)
Total	199 (100%)	197 * 4.5(1) = 884	2 * 12.0(2) = 24	908 (823–994)(3)

(1) Correction factor screen-positive group: total number of screen-positives divided by number of screen-positives in the study sample (4592/1024 = 4.5).

(2) Correction factor screen-negative group: total number of screen-negatives divided by number of screen-negatives in the study sample (6085/506 = 12.0).

(3) 95% Confidence Interval.

In the first step, we split our sample into four groups; no use of an antidepressant, justified use, possibly justified use and unjustified use. We will refer to these groups as “justification groups”. After that, we registered the number of screen-positives and screen-negatives in each of the justification groups. These numbers were then multiplied by a correction factor (respectively total screen-positives divided by screen-positives in sample (4592/1024) or total screen-negatives divided by number of screen-negatives in our sample (6085/506)) to calculate the estimated number of persons from each justification group in the original screen-positive and screen-negative groups. Finally, we added up the estimated numbers screen-positives and negatives for each justification group.

A 95% confidence interval (95% CI) was calculated for all estimated numbers. This was done by first calculating the standard error of the proportion with the proper mathematical formula. This number was then multiplied by 1.96 and subtracted and added to the proportion in order to get the 95% CI of the proportion, which could then be recalculated to the 95% CI of the absolute number by multiplying by n.

We used a Chi square statistic to test for significant differences in justification for an antidepressant between antidepressant users with no/mild/moderate/severe MDD.

All statistical calculations were performed using SPSS for Windows Release 16.0.

## Results

### Study population

The average age of the population was 45.8 years, 1054 (68.8%) of respondents were female and 477 (31.2%) male.

The diagnoses of the respondents with lifetime depression/depressive symptoms (n = 1064, of whom 651 with a comorbid anxiety disorder) were as follows: 807 respondents had a lifetime MDD of whom 428 in the past year; 23 had a lifetime Dysth without history of MDD of whom 16 in the past year; and 234 had a lifetime miD or depressive symptoms in lifetime without a history of MDD or Dysth, of whom 44 had a miD in the past month (incidence in last year unknown). The diagnoses of the respondents with a lifetime anxiety disorder (n = 762) were: 345 patients with social phobia; 344 patients with a panic disorder (with or without agoraphobia); 131 patients with agoraphobia; and 330 patients with a generalized anxiety disorder (324 patients had more than one anxiety disorder).

### Justified and non-justified treatment

Of the respondents with a depression/depressive symptoms (MDD, dysthymia, miD or depressive symptoms, n = 1064), 189 (17.8%) used an antidepressant, of whom 75 (39.7%) had a justification, 68 (36.0%) a possible justification and 46 (24.3%) did not have a justification.

However, of the 46 antidepressants users with a depression/depressive symptoms without a justification for treatment with an antidepressant (non-recurrent MDD, miD or depressive symptoms) nine had only a depression/depressive symptoms and 37 had a lifetime anxiety disorder (26 in the past year), which means that the antidepressant may have been prescribed for the anxiety disorder rather than the depression. This means that in only nine patients (4.5%) with a depression/depressive symptoms treatment was not justified.

Of the 111 respondents with a lifetime anxiety disorder without a depression/depressive symptoms eight patients (10.1%) used an antidepressant, in three this was justified and in five possibly justified.

Only 2 (0.6%) of the 356 respondents without a lifetime depression or anxiety disorder used an antidepressant. This treatment obviously was not justified.

Table 2 shows a summary of justified and not justified treatment in our sample. In total 11 respondents used an antidepressant without a definite or possible justified reason. Eight of them used a SSRI, one a TCA (at low dose) and two another antidepressant; the duration of antidepressant use varied from 0 to 120 months, with a median of 48 months. Nine of these respondents had a depression/depressive symptoms (six a single episode of MDD more than one year ago, three miD or depressive symptoms). They represent 0.77% of the 1175 respondents with a lifetime depression or anxiety disorder. The other two represent 0.56% of the 356 respondents without a depression/depressive symptoms or anxiety disorder. Thus, 6 of the 11 respondents (54,5%) had a justified reason for treatment with an antidepressant at some earlier point in their life because of an episode of MDD.

**Table 2:** Treatment with antidepressants and justification for treatment.

Justification for treatment	No AD	AD	Total
Unjustified	566 (98.1%)	11 (1.9%)	577
Possibly justified	225 (70.8%)	93 (29.3%)	318
Definitely justified	541 (85.1%)	95 (14.9%)	636
Total	1332 (87.0%)	199 (12.9%)	1531

Treatment was considered definitely justified in case of a MDD in the past year, or recurrent or chronic MDD with antidepressant treatment ≤24 months, or an anxiety disorder in the past year.

Treatment was considered possibly justified in case of dysthymia in the past year, or a recurrent or chronic MDD with antidepressant treatment for more than 24 months, or an anxiety disorder over one year ago.

All other antidepressant treatment was considered unjustified.

All numbers are absolute number of respondents (percentage of total respondents in row).

AD = antidepressant.

Table 3 shows the relation between severity of depression at baseline and antidepressant use. This table shows that antidepressant users with a mild (recurrent) MDD are less often classified as definitely justified AD use. We performed a Chi square test comparing the justification groups for antidepressant users with moderate to severe (recurrent) MDD to antidepressant users with no MDD or mild (recurrent) MDD. The difference was significant (p = 0.015), antidepressant users with moderate to severe (recurrent) MDD more often had a justified reason for the use of an antidepressant compared to antidepressant users with no MDD or mild (recurrent) MDD.

**Table 3:** Severity of depression at baseline and indication for treatment with an antidepressant, based only on depression diagnosis.

	Definitely justified AD users (n,%)	Possibly justified AD users (n,%)	Unjustified AD users (n,%)	Total (n)
MDD Single Mild	7 (24%)	6 (21%)	16 (55%)	29
MDD Recurrent Mild	12 (39%)	19 (61%)	N/A	31
MDD Single Moderate	19 (54%)	7 (20%)	9 (26%)	35
MDD Recurrent Moderate	9 (64%)	5 (36%)	N/A	14
MDD Single Severe	11(33%)	8 (24%)	14 (42%)	33
MDD Recurrent Severe	17 (53%)	15 (47%)	N/A	32
Dysthymia	N/A	8 (100%)	0 (0%)	8
miD/depressive symptoms	N/A	N/A	7 (100%)	7
Total	75	68	46	189

N/A =  not applicable.

Respondents with a recurrent MDD and possibly justified AD use had a possible justification because they had used an antidepressant for more than 2 years.

### Use of antidepressants in the primary care population

With the selection procedure as described in the method section, we can calculate what our findings mean for the total population of respondents (n = 10,677) who returned the K-10 plus (table 1).

In our sample there were 188 antidepressant users with justified antidepressant use of whom 95 (50.5%) with a definite justified reason, and 93 (49.5%) with a possible justified reason: 2 among the respondents with a negative K-10 plus (n = 6085, of whom 506 participated in the study) and 186 among the respondents with a positive K-10 plus (n = 4592, of whom 1023 participated). Recalculated to the respondents who completed the K-10, there are 24 (95% CI (0 to 57) antidepressant users with a definite or possibly justified reason in the screen-negative group and 835 (95% CI 726 to 943) in the screen-positive group.

All 11 respondents who used an antidepressant without a definite or possible justified reason had a positive K-10 plus. The total number of antidepressant users without a justified reason among all respondents with a positive K-10 plus can be recalculated at 49 (95% CI 20 to 78). As there were no antidepressant users without indication with a negative K-10 plus, the total number of respondents who used an antidepressant without indication among all 10,677 people who completed the K-10 plus, is also 49 (95% CI 20 to 78). K-10 plus results were unknown for one respondent without antidepressant use.

Combining the results of antidepressant users with and without justification, recalculated to the sample of 10,677 GP patients from 65 GPs who had consulted their GP in the past four months irrespective of reason for consultation and did return a completed screening questionnaire, 908 (8.5%), (95% CI 823 to 994), used an antidepressant of whom 859 (94.6% of the antidepressant users) with a definite (n = 434, 47.8%) or possible (n = 425, 46.8%) justification in accordance with the guideline and 49 patients (5.4% of the antidepressant users) without justification.

## Discussion

### Summary of main findings

The main finding of our study is that overtreatment with antidepressants did not appear to be a very frequent problem in Dutch primary care. Of all GP patients (and after excluding patients who were treated in secondary care including hospitals, institutes for mental health care and psychiatrists with private practices) 8.5% received an antidepressant. When compared with the guidelines of the Dutch General Practitioner's Association (NHG), 94.6% of the antidepressant use was in accordance with the guideline (47.8% definitely and 46.8% possibly) while only 5.4% was not. The latter was for the large part not due to treatment of mild forms of depression or patients without psychiatric diagnoses, but to non-justified long continuation of antidepressant treatment in patients who at some earlier point in their life had a justified reason for treatment with an antidepressant.

### Strengths and limitations of the study

The current study has several very strong points. First, we used a screening method to recruit participants which did not affect the awareness of patient's psychiatric status for GPs in our study. This means that the GPs could only rely on their own diagnostic judgments also for their prescription of antidepressants. The second strength of this study is its large sample size, which is rather rare in a primary care study. The third strength is that all patients were diagnosed based on a structured interview and not on the GPs' records.

However there are also limitations. First, the last mentioned strength is also a weakness, as the structured interview we used (the CIDI) does not assess the degree of suffering and dysfunction, which should be part of the GPs' consideration for antidepressant treatment according to the guideline recommendations. Second, the representativeness of the population may be limited. The GPs in this study, and thus their patients, may not be representative for all Dutch primary care practices, as these practices/GPs agreed to participate in the NESDA study, and thus have interest in psychiatric research. This may be associated with a better compliance to the guidelines for depression and for anxiety disorders. Next to that, according to the SFK about 760,000 Dutchmen were prescribed an antidepressant (about 6.3% of the adult population) in the last 6 months of 2005. In our primary care sample we recalculated that 8.5% used an antidepressant. This higher percentage may be explained by the fact that respondents were selected among the patients who consulted their GP in the last four months. The non-response to the screening questionnaire did not seem to be biased with regard to psychopathology.[33]

Fourth, we did not have access to the full electronic patient file from the GPs. Therefore we did not know why they prescribed an antidepressant. This might have been of interest, as antidepressants can also be prescribed for other indications than depression or anxiety disorder. Some antidepressants including TCAs at low dose for example are used for neuropathic pain. However, if any effect, this would result in an ever lower estimation of overtreatment with antidepressants in our sample. In addition, we could not determine the ground on which the GPs based their treatment decisions, therefore we could not determine if a decision to (dis)continue an antidepressant was according to guideline recommendations. This applies especially to the category ‘possibly justified’ in patients who had recovered from a recurrent depressive episode more than two years ago, as we do not have information as to why the antidepressant was continued in these patients. Among them are definitely patients who continue their antidepressants for good reasons, e.g. patients who had stopped their antidepressant after a recurrent episode and who developed a new recurrence, and patients who tapered off and subsequently developed minor symptoms and therefore restarted medication. This limitation also means that we were unable to estimate the severity of symptoms at the time the antidepressant was started. Table 3 showed that some respondents with a mild episode of MDD received treatment with antidepressants. The guideline at time of the study does not differentiate between mild and moderate to severe depressive episodes in its recommendations, but more recent guidelines do. For example the new Dutch multidisciplinary depression guideline is much more conservative and recommends to reserve treatment with antidepressants for patients with more severe depressive states.[42] It is unclear whether the patients with mild symptomatology at baseline had more severe symptomatology at the time the antidepressant treatment was initiated.

A final limitation is the cross-sectional design of the study, as a result of which the start of symptoms and time of remission could not be determined precisely. We allowed treatment with antidepressants in case of a MDD in the past year. In some cases treatment probably should have been stopped before the interview, because the patient had been in remission for (more than) 6 months. Because of the cross-sectional design, we also had to rely on recall of symptoms of depression and anxiety during the past year and for lifetime diagnoses. Several researchers have questioned the reliability of retrospective recall of symptoms during a single interview in persons with a history of depression.[43], [44]

### Comparison with existing literature

Several previous studies also looked at rates of overtreatment, with various outcomes. The major contrast with our study is that these studies only looked at relative small groups of GP patients and did not allow recalculation of the results to the total population of GP patients. Two of the previous studies found high rates of overtreatment: 25% (Sihvo et al.) and even 35% (Berardi et al.).[17], [18] Berardi et al. described a group of 361 primary care patients of whom 82 used an antidepressant. They only considered treatment with antidepressants indicated for current depression, ignoring possible continuation or maintenance treatment and other indications for the use of antidepressants like anxiety disorders. This is a rather limited definition, as continuation treatment is a well-established part of depression treatment and many antidepressants are also registered for anxiety disorders. Sihvo et al. described a group of 526 patients who used an antidepressant. They adopted a definition of overtreatment slightly broader than ours. Treatment was considered “non-psychiatric” in case there was no CIDI diagnosis of MDD, Dysth, anxiety disorder (generalised anxiety, social phobia, panic disorder or agoraphobia), bipolar disorder or alcohol dependence in the last 12 months.

The third previous study (Cameron et al) reported a small percentage (exact percentage not mentioned) of overtreatment in a Scottish primary care sample of 120 antidepressant users.[19] They did not have a diagnosis based on a structured interview, but used a cutt-off score of the Hospital Anxiety and Depression Scale (HADS). Of their sample 45 had “no depression”, 34 had a “possible depression” and 41 had “probable depression.” In the respondents with “no depression”, they reported that 32 had a history of depression (as recorded by the GP), 5 had anxiety disorder, 5 had neuropathic pain and for only 3 it remained unclear why they received an antidepressant.

It can be argued that our definition of non-justified antidepressant use is rather small, and that in reality more patients in our sample did not actually need an antidepressant. First, we defined lifelong treatment non-justified only for patients without a definite or possible justification. This created the problem of how to classify the category ‘possible justified’ for treatment beyond two years of patients with a recurrent depression, while most guidelines recommend several years and only for a (non-specified) subgroup lifelong treatment. Second, also in case of anxiety disorders it could be argued that lifelong treatment is unnecessary in many if not most cases. Third, the diagnosis chronic MDD was based on the self-report of 24 months (of probably uninterrupted) symptoms of depression in the past five years according to the life chart and a lifetime diagnosis of MDD. It could very well be that these patients had no chronic MDD, but just symptoms of depression during two or more years. This would mean that in more patients antidepressants would be unnecessary. When we would have classified all possible justified cases as overtreatment the percentage would indeed rise substantially: from 5.4% to 52.2%, which is much higher than Berardi et al. and Sihvo et al. and clearly illustrates the importance of a clear definition of overtreatment. We think however that our definition including life long treatment for patients with recurrent depressive episodes is justifiable, considering the high recurrence rate of depression, especially after multiple episodes.[45], [46] As discussed above, at least part of the respondents with a possible justification will have a very good reason for the use of an antidepressant. Moreover, there could be patients without a justified reason for the use antidepressants, for whom the physician (or the patient himself) found an antidepressant needed, e.g. because of residual symptoms after a single episode of MDD. One could also argue that, although not indicated according to the guideline, this is justified treatment.

If indeed the percentage of overtreatment is lower in the Netherlands than in other countries: what would be the explanation? A possible explanation is the difference in primary care systems. In the Netherlands, the GP is the gatekeeper to secondary and mental health care; patients need a reference from the GP before they can consult mental health care. Secondly, Dutch GPs have been trained during the last years in how to diagnose and treat depression and anxiety disorders, as part of the implementation of the Dutch primary care guidelines. This also implies that our results probably cannot be generalized globally, as primary healthcare systems vary across countries.

Another explanation for the difference could be that antidepressant use in our study was based on drug-container inspection in most patients and on self-report in a minority of cases, while the Dutch Foundation for Pharmaceutical Statistics (SFK, www.sfk.nl) data are based on pharmacy prescription data. From previous studies it is known that many patients do not pick up their prescription or do not take their medication.[47], [48]

In this study we focused on overtreatment with antidepressants, and therefore we looked at whether patients received an antidepressant without a justified reason. However, “justified” does not mean “needed”. Patients with a mild or even moderately severe episode of MDD do not necessarily need treatment (either an antidepressant or psychotherapy), although they do have a justified reason for treatment with an antidepressant. New guidelines like the recent 2009 update of the Dutch multidisciplinary guideline for depression recommend to reserve antidepressants for patients with moderate to severe depression.[42] Therefore, an alternative interesting question is: who needs an antidepressant, and how many of the patients who need an antidepressant do actually receive an antidepressant. This however, was not the focus of our study as undertreatment of depression has already been the focus of many studies in the past.[11]–[16] Moreover, the NESDA study is not suitable for answering this question. It is a naturalistic study and part of the study population did not seek any help. It is therefore impossible to determine which patients are “undertreated” by their GP and which did not seek help for their psychological complaints.

### Implications for future research and clinical practice

In conclusion, the current study provides a unique insight into the justification of the prescription of antidepressants in Dutch primary care. In contrast to the scarce literature, the rate of overtreatment with antidepressants in the present study was low. Another interesting finding is that overtreatment is not so much due to treatment of mild forms of depression or patients without psychiatric diagnoses, but rather to an excessive duration of antidepressant treatment in patients with remitted (recurrent) MDD. This latter finding presents several implications for clinical practice. First, projects on optimizing treatment with antidepressants in primary care, should not focus on reducing overtreatment but on identifying patients who do not need antidepressants anymore. Second, GPs should be aware that apparently many patients tend to continue the antidepressant over many years. Which of these patients might be able to stop the antidepressant is unclear. Therefore, further studies addressing this question are warranted before starting campaigns to reduce the use of antidepressants in primary care.
